# Supplementary material for: Prenatal Octamethylcyclotetrasiloxane Exposure Impaired Proliferation of Neuronal Progenitor, Leading to Motor, Cognition, Social and Behavioral Functions
Source: Int J Mol Sci. 2021 Nov 30;22(23):12949. doi: 10.3390/ijms222312949 (PMC8657511; doi:10.3390/ijms222312949)
Supplement: Supplementary file 1 [file ijms-22-12949-s001.zip › ijms-1473035-supplementary.pdf]

International Journal of
Molecular Sciences

MDPI

Prenatal Octamethylcyclotetrasiloxane Exposure Impaired Proliferation of Neuronal Progenitor, Leading to Motor, Cognition, Social and Behavioral Functions

Dinh Nam Tran [1], Seon-Mi Park [1], Eui-Man Jung [2] and Eui-Bae Jeung [1,*]

Supplementary material: Supplemental Methods, Supplementary Figures S1–S3 and Legends

Supplemental Methods

Behavioral analysis

All experiments were performed between 8:00 AM and 2:00 PM in an empty testing room within the animal vivarium. On testing days, mice were habituated in the test room for at least 1 h before the start of testing and the testing was performed by laboratory technicians blinded to the mouse group information. A test−free period of 2 days to 1 week was used between two consecutive tests. All experimental areas were cleaned with 70% ethanol before the tests and between subjects. The animal number is reported in Figure legends and Supplementary Table 1.

Open field test. Open field consisted of a large acrylic cube measuring 50 cm tall, 60 cm wide with a white bottom and mice were automatically video−tracked using EthoVision XT14 software (Noldus Information Tech Inc, Leesburg, VA, USA). Mice were individually placed near the wall−side and locomotor behavior was recorded for 5 min. Time spent in the center zone (15 x 15 cm), velocity and distance traveled was evaluated.

Morris water maze test. Spatial learning and memory were measured using a circular pool (90 cm diameter, 40 cm high), filled with water (25±1°C) and the water was made opaque by adding skim milk. The tank was and divided into four equal quadrants (Q1−4) by lines drawn on the floor. Mice were trained to locate a platform (10 cm diameter, 20 cm high) made of plexiglass was placed in the middle of a Q3 (12 cm from the pool's edge and 1 cm below the surface of the water) with visual cues on the pool walls as spatial references. Over a period of 9 days (cue training (4 days) and followed by spatial training (5 days), mice received four training trials with an intertrial interval of 3 to 5 min. Each trial ended when the mice had reached the submerged platform within 60 s. If the mice failed to reach the platform within 60 s, the experimenter gently guided the mice to the platform allowed to rest on it for 30 s. The mean of escape latencies (second) for 4 trials is represented for the learning result for each mouse. Following the completion of spatial learning, spatial memory was measured using a 60 s probe trial (with the platform was removed from the pool) 24 h following completion of the last training session. Video recordings of the probe trial were analyzed using EthoVision XT14 and spatial memory was scored as the time spent in the target platform where the platform was positioned during training.

Novel object recognition test. A test mouse was placed into an empty open field arena and allowed to explore freely during a 5−minute habituation period. The following day, in the familiarization session, the subject mice were placed into the open field arena in the presence of two identical objects (2 cm width× 5cm length× 9 cm tall in opposite corners of the arena, 7cm from the side walls) and freely explored for 10 min. The mouse was returned to its home cage, and after 6 hours, one of the objects was replaced by a novel object (different shape and color compared to the old object). The subject mice were again introduced in the arena and allowed to explore for another 10 min. The duration of time mice spent exploring with familiar object vs novel object (sniffing or exploring at distance within 2 cm of the object) were recorded. Videos were analyzed using EthoVision XT14.

Rotarod test. Motor coordination was assessed in an accelerating rotarod test (4–40 r.p.m.) using a Rota Rod (Panlab, Sydney, Australia). Mice were introduced in a rotarod apparatus rotating drums (3cm in diameter) and measuring retention time on the rod. Mice were given a 3−day training period with the speed of the rotarod accelerating from 4 to 40 rpm over 10 minutes. The following a 7−day testing period was conducted (day: 4, 6, 8, 10, 14, 16, and 20) with the speed of the rotarod accelerating from 4 to 40 rpm over 5 minutes. Three trials per day were conducted. At least 20min recovery time was allowed between trials.

Hanging-wire grip test. A hanging−wire grip test apparatus was performed to assess balance and grip strength. The apparatus consists of a box (42 × 42 × 42 cm) with a wire−mesh grid (20 × 20 cm) on top that can be inverted. Mice were placed on the wire mesh, which was then inverted, causing the animal to grip the wire. The latency to the mouse falling was recorded, with a 90 s cutoff time and scored as follows: 1, falls off within 10 s; 2, falls off within 11 − 25 s; 3, falls off within 26 − 60 s; 4, falls off within 61 − 90 s; 5, falls off after 90 s. Mice were individually assessed and the mean score of each group was determined.

Three-chamber social test. The three-chambered apparatus consisted of three Plexiglas chambers, each chamber was 20 cm width× 40 cm length× 22 cm tall, and the dividing walls were made from clear Plexiglas, with small rectangular openings (5 × 3 cm) allowing access into each chamber. Both side chambers contained a cylindrical plastic cage (17 cm in height, a bottom diameter of 8 cm with the bars spaced 1 cm apart) in the corner that used to hold the stranger mice. First, the subject mouse was placed in the middle chamber and allowed to freely explore all three chambers with an empty plastic cage in each side chamber for the 5 min habituation period. For sociability testing, an unfamiliar same age, sex, and weight−matched (within 5 g) mouse was placed in a cylindrical plastic cage in one of the side chambers (Stranger 1- 'S1'), and an empty cylindrical plastic cage (Empty - 'E') on the other side−chamber. Then, the subject mouse was placed in the center chamber and allowed to freely explore all three chambers for 10 min while being video recorded. For the social novelty test, a wild−type stimulus mouse (Stranger 2− 'S2') was placed in the empty plastic cage and the subject mouse again freely explored all three chambers for 10 min. The time spent in each chamber was video−tracked using Ethovision XT14 software. The preference index for each animal was calculated as: Preference index = $\frac{(S1-E)}{(S1+E)}$ or as $\frac{(S2-S1)}{(S2+S1}$: where 'E', 'S1' and 'S2' are the time spent in close proximity with empty cage, the stranger animal 1 and 2, respectively.

Forced swimming test. A mouse was gently placed into a glass cylinder (20 cm height, 17 cm diameter) filled with water to a depth of 12 cm at 25°C. All mice were forced to swim for 5 min and then were removed from the water, dried, and returned to their home cages. 24 h later, the mice were again placed in the cylinder, and acclimatized for 1 minute, followed by a testing period of 5 minutes in which the duration of immobility was measured. Videos were analyzed using EthoVision XT14.

Marble burying test. Mice were individually placed in a Plexiglas cage (25 cm width × 40 cm length × 18 height cm) containing 5 cm−deep fresh, unscented mouse bedding material (corn cob bedding) for 10 min before the test. Then the mouse was removed from the testing cage and 20 glass marbles (15 mm diameter) were carefully overlaid equidistantly from each other in a 4 × 5 arrangement on the surface of the bedding. The mouse was placed into a corner of the cage and allowed to bury marbles for 30 min. The mouse was removed from the cage and the marbles were imaged and scored by a scorer blinded to the conditions. A marble was considered buried if more than 75% marble was covered by the bedding.

Grooming test. Mice were placed in a separate clear plastic cage (17×32×14cm). Self−grooming behavior was recorded for 10 min. Self−grooming was defined as scratching of face, head, or body with the two forelimbs, or licking body parts. Cumulative time spent grooming was scored for each mouse.

Nesting test. Mice were individually placed in its one clean cage with a nestlet of tissue cotton (5× 5 cm, mean weight 2.5 g) at 7:00 AM and recorded 12 h later. Nest quality produced by each mouse was assessed according to a 5−point rating scale from 1 to 5 as follows [1]. All mice were assessed together and the mean score of each group was determined.

Supplemental Reference

1. Deacon, R.M., Assessing nest building in mice. *Nature protocols.* **2006**, 1, 1117-1119.

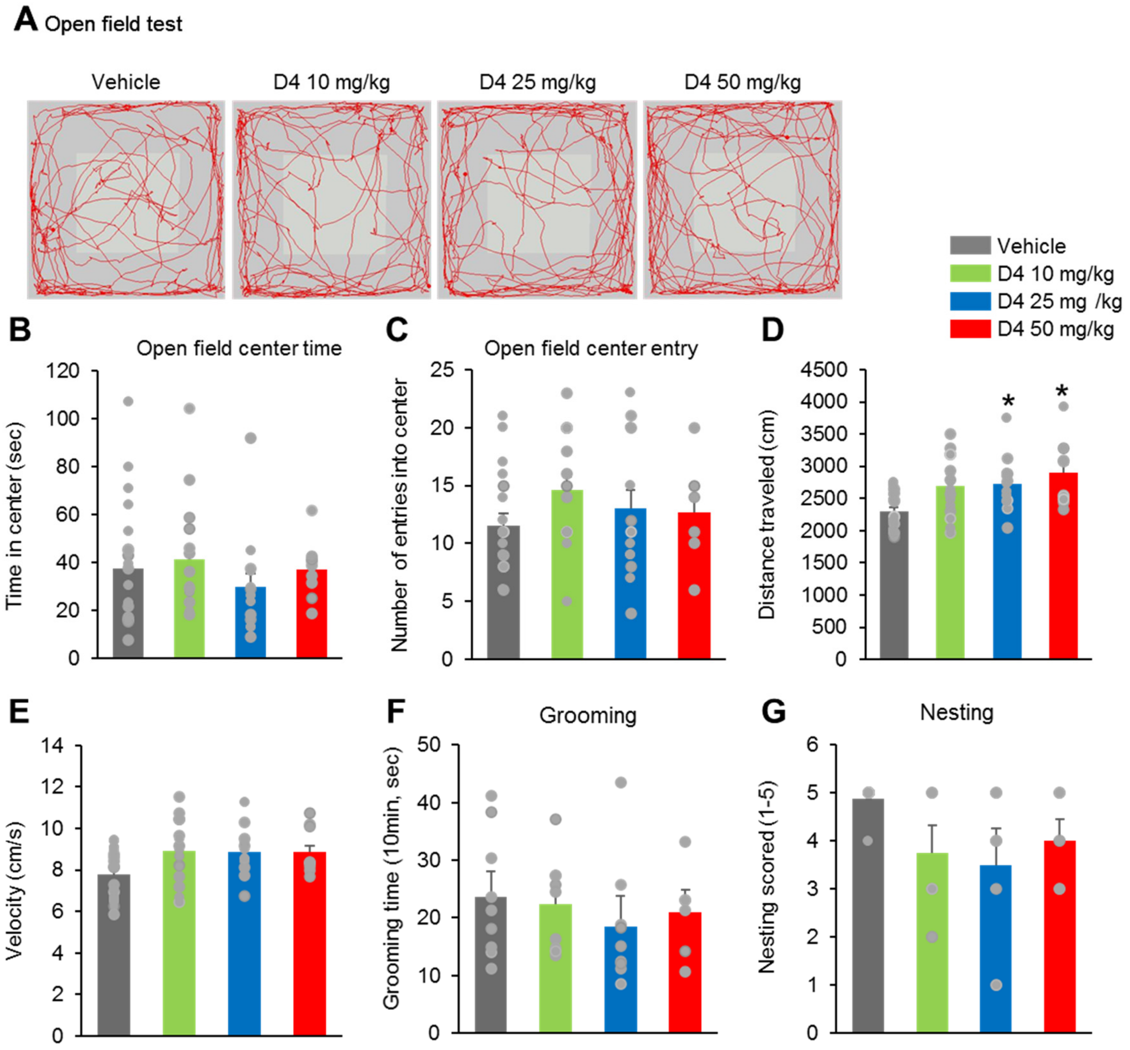

Supplementary Figure S1. Prenatal D4 did no elevates anxiety−like behavior. (**A**) Representative tracing of mouse travel in the open field test. (**B, C, D, E**) Quantification of (**A**). D4−treated groups displayed no difference in both open field center time, open field center entry and velocity compared to vehicle group. However, D4 25 mg/kg and D4 50 mg/kg groups showed increased in the moving distances (**F, G**) In grooming and nesting test, D4-treated groups also showed no significant. Data represent mean ± SEM. Statistical significance was determined by one−way ANOVA with Bonferroni correction. $^{*}p < 0.05$ vs. vehicle

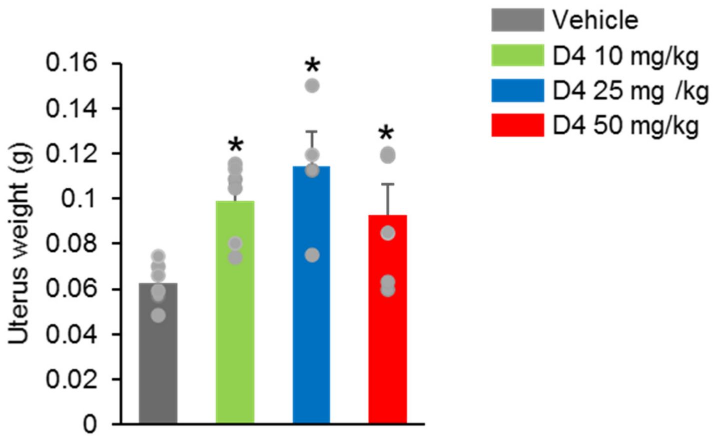

Supplementary Figure S2. D4 increased uterine weight. D4−treated groups exhibited slightly higher in the uterine weight than in those of Vehicle group ($F_{3,21} = 6.731$, $p = 0.0019$). n = 6 mice for vehicle, 6 mice for D4 10 mg/kg, 4 mice for D4 25 mg/kg, 7 mice for D4 50 mg/kg. Data represent mean ± SEM. Statistical significance was determined by one−way ANOVA with Bonferroni correction. $^{*}p < 0.05$ vs. vehicle.

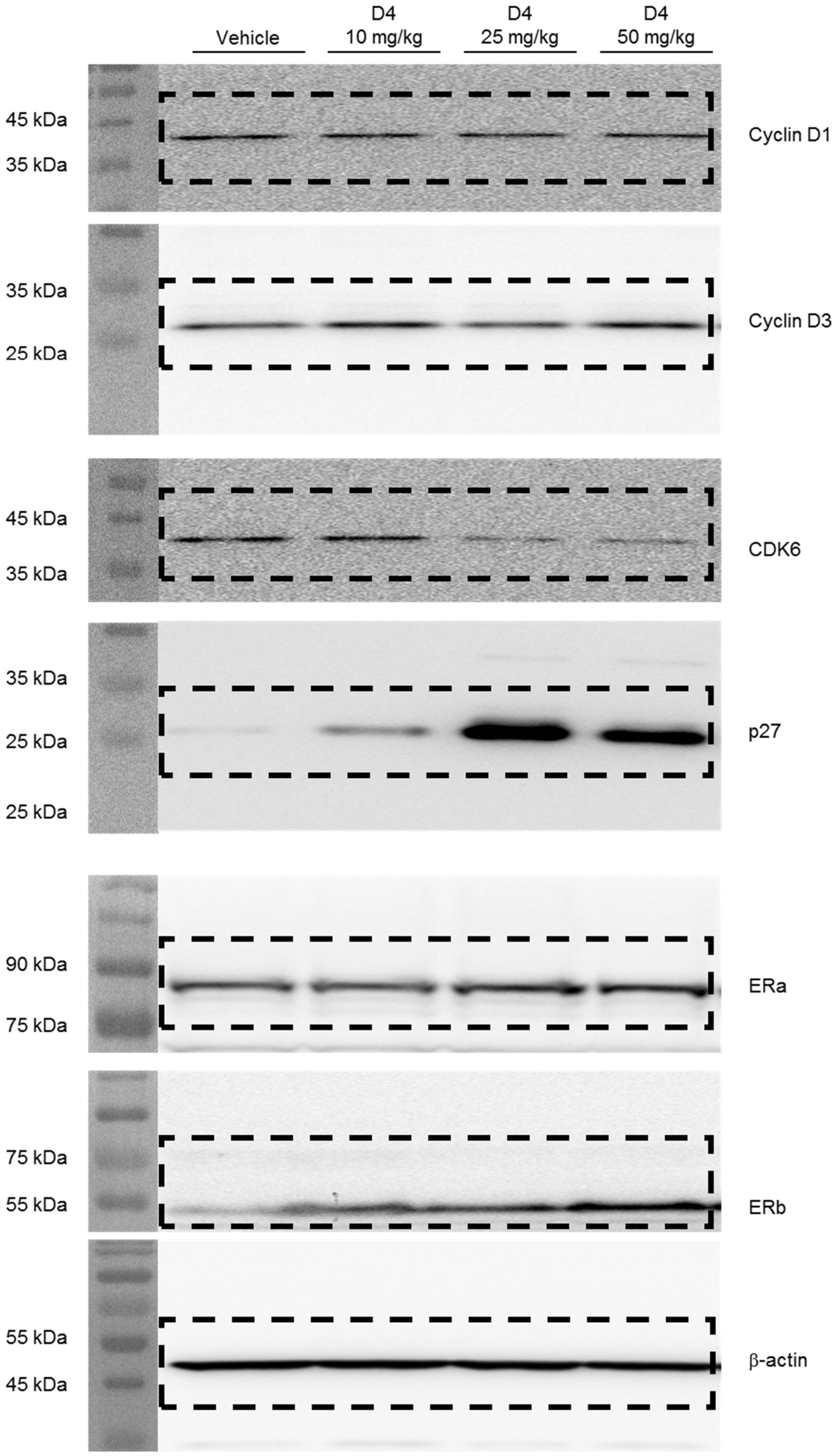

Supplementary Figure S3. Full length Western blots.

Full length Western blots for cropped images in Figure 6A

Supplementary Table S1. Behavior statistic data.

Test	Duration	Measurement	Number of animals	Mean ± SEM values		Statistical test: Test	Statistical test: Test statistic value	Statistical test: p value
Morris water maze	1 min	Escape latency (sec)	VE = 10<br>D4 10 = 11<br>D4 25 = 11<br>D4 50 = 10	Day1	*VE* = 51.16 ± 2.52<br>*D4 10* = 56.43 ± 1.81<br>*D4 25* = 52.84 ± 2.44<br>*D4 50* = 54.04 ± 1.49	One–way ANOVA with Bonferroni correction test	$F_{3,41}$ = 0.7631	0.5227
				Day2	*VE* = 28.67 ± 2.82<br>*D4 10* = 32.94 ± 3.21<br>*D4 25* = 33.21 ± 3.57<br>*D4 50* = 46.05 ± 2.81		$F_{3,41}$ = 3.090	0.0174
				Day3	*VE* = 19.59 ± 1.99<br>*D4 10* = 25.69 ± 2.98<br>*D4 25* = 30.34 ± 3.54<br>*D4 50* = 37.04 ± 7.39		$F_{3,41}$ = 4.625	0.0014
				Day4	*VE* = 15.61 ± 1.54<br>*D4 10* = 19.36 ± 2.67<br>*D4 25* = 25.83 ± 3.45<br>*D4 50* = 30.25 ± 3.56		$F_{3,41}$ = 6.197	0.0001
				Day5	*VE* = 9.00 ± 0.58<br>*D4 10* = 11.55 ± 1.53<br>*D4 25* = 17.56 ± 2.48<br>*D4 50* = 28.36 ± 3.84		$F_{3,41}$ = 9.697	< 0.0001
				Day6	*VE* = 9.44 ± 0.62<br>*D4 10* = 10.64 ± 0.99<br>*D4 25* = 16.77 ± 2.56<br>*D4 50* = 26.23 ± 3.15		$F_{3,41}$ = 10.51	< 0.0001
				Day7	*VE* = 6.27 ± 0.90<br>*D4 10* = 7.42 ± 1.13<br>*D4 25* = 10.45 ± 1.96<br>*D4 50* = 22.04 ± 3.62		$F_{3,41}$ = 7.780	< 0.0001
				Day8	*VE* = 4.25 ± 0.42<br>*D4 10* = 5.59 ± 0.71<br>*D4 25* = 6.68 ± 0.86<br>*D4 50* = 16.33 ± 3.12		$F_{3,41}$ = 7.534	< 0.0001
				Day9	*VE* = 5.14 ± 0.64<br>*D4 10* = 5.48 ± 0.67<br>*D4 25* = 5.45 ± 0.78<br>*D4 50* = 18.33 ± 3.54		$F_{3,41}$ = 9.826	< 0.0001
		Platform crossing		*VE* = 5.86 ± 0.70<br>*D4 10* = 4.94 ± 0.52<br>*D4 25* = 4.28 ± 0.54<br>*D4 50* = 2.60 ± 0.56			$F_{3,38}$ = 3.473	0.5227
		Distance move (cm)		*VE* = 562.80 ± 41.05<br>*D4 10* = 660.67 ± 42.30<br>*D4 25* = 521.44 ± 41.70<br>*D4 50* = 506.72 ± 68.67			$F_{3,38}$ = 2.267	0.0855
		Velocity (cm/s)		*VE* = 9.17 ± 0.67<br>*D4 10* = 10.84 ± 0.69<br>*D4 25* = 8.28 ± 0.71<br>*D4 50* = 7.02 ± 1.23			$F_{3,38}$ = 2.389	0.0733
		Time spent in platform (s)		*VE* = 5.53 ± 0.62<br>*D4 10* = 4.13 ± 0.42<br>*D4 25* = 2.21 ± 0.53<br>*D4 50* = 2.96 ± 0.40			$F_{3,38}$ = 3.252	0.0365

Rotarod	5 min	Latency to fall (sec)	VE = 10<br>D4 10 = 10<br>D4 25 = 10<br>D4 50 = 10	Day1	*VE* = 76.95 ± 13.13<br>*D4 10* = 36.29 ± 5.04<br>*D4 25* = 38.08 ± 6.47<br>*D4 50* = 21.38 ± 3.56	One−way ANOVA with Bonferroni correction test	$F_{3,39}$ = 2.992	0.0492
				Day2	*VE* = 113.21 ± 7.67<br>*D4 10* = 68.17 ± 10.00<br>*D4 25* = 74.58 ± 6.88<br>*D4 50* = 49.76 ± 6.42		$F_{3,39}$ = 6.578	< 0.0001
				Day3	*VE* = 120.67 ± 7.13<br>*D4 10* = 107.58 ± 11.34<br>*D4 25* = 112.63 ± 11.62<br>*D4 50* = 70.81 ± 6.79		$F_{3,39}$ = 6.674	< 0.0001
				Day4	*VE* = 93.26 ± 5.16<br>*D4 10* = 91.33 ± 7.55<br>*D4 25* = 63.20 ± 5.66<br>*D4 50* = 59.86 ± 5.01		$F_{3,39}$ = 6.280	0.0001
				Day6	*VE* = 103.74 ± 5.11<br>*D4 10* = 97.42 ± 5.52<br>*D4 25* = 72.08 ± 6.42<br>*D4 50* = 64.95 ± 6.30		$F_{3,39}$ = 8.249	< 0.0001
				Day8	*VE* = 110.74 ± 6.04<br>*D4 10* = 98.56 ± 3.72<br>*D4 25* = 85.58 ± 6.83<br>*D4 50* = 76.42 ± 5.11		$F_{3,39}$ = 4.188	0.0035
				Day10	*VE* = 116.39 ± 6.70<br>*D4 10* = 103.71 ± 8.72<br>*D4 25* = 88.00 ± 5.40<br>*D4 50* = 77.66 ± 4.62		$F_{3,39}$ = 3.700	0.0074
				Day14	*VE* = 111.63 ± 5.04<br>*D4 10* = 108.63 ± 8.75<br>*D4 25* = 82.35 ± 6.03<br>*D4 50* = 76.75 ± 5.07		$F_{3,39}$ = 7.045	< 0.0001
				Day16	*VE* = 108.28 ± 5.35<br>*D4 10* = 106.04 ± 8.54<br>*D4 25* = 82.00 ± 7.08<br>*D4 50* = 76.05 ± 3.74		$F_{3,39}$ = 6.191	0.0001
				Day20	*VE* = 106.62 ± 3.08<br>*D4 10* = 110.95 ± 6.16<br>*D4 25* = 83.60 ± 7.54<br>*D4 50* = 76.76 ± 3.78		$F_{3,39}$ = 8.378	< 0.0001
Novel object recognition	10 min	Recognition index (%)	VE = 14<br>D4 10 = 12<br>D4 25 = 12<br>D4 50 = 10		*VE;* Familiar = 41.67 ± 6.80<br>*VE;* Novel = 58.33 ± 6.80	Two−tailed Student's *t* test	*t* = 3.240	0.0033
					D4 10; Familiar = 44.61 ± 4.58<br>*D4 10;* Novel = 55.39 ± 4.58		*t* = 2.886	0.0086
					D4 25; Familiar = 55.28 ± 2.72<br>*D4 25;* Novel = 44.72 ± 2.72		*t* = 2.040	0.0687
					D4 50; Familiar = 48.28 ± 3.47<br>*D4 50;* Novel = 51.72 ± 3.47		*t* = 0.780	0.4576
Grip strength	1 min 30 second	Scoring	VE = 14<br>D4 10 = 14<br>D4 25 = 14<br>D4 50 = 14		*VE* = 4.86 ± 0.22<br>*D4 10* = 4.86 ± 0.22<br>*D4 25* = 4.79 ± 0.29<br>*D4 50* = 4.93 ± 0.13	One−way ANOVA with Bonferroni	$F_{3,55}$ = 0.2382	0.9157

						correction test		
Social behavior	10 min	Time in chamber	VE = 10<br>D4 10 = 10<br>D4 25 = 10<br>D4 50 = 10	Sociability	*VE*; Empty = 175.07 ± 12.16<br>*VE*; Stranger I = 242.52 ± 10.65	Two−tailed Student's *t* test	*t* = 3.558	0.0031
					D4 10; Empty = 208.73 ± 12.90<br>*D4 10*; Stranger I = 254.93 ± 14.16		*t* = 2.182	0.0443
					D4 25; Empty = 167.94 ± 7.11<br>*D4 25*; Stranger I = 242.99 ± 6.46		*t* = 6.230	< 0.0001
					D4 50; Empty = 170.23 ± 9.65<br>*D4 50*; Stranger I = 284.01 ± 13.64		*t* = 4.288	< 0.0001
				Social novelty	*VE*; Stranger I = 150.70 ± 17.66<br>*VE*; Stranger II = 241.37 ± 22.69		*t* = 2.516	0.0271
					D4 10; Stranger I = 184.64 ± 13.02<br>*D4 10*; Stranger II = 256.62 ± 11.19		*t* = 3.793	0.0016
					D4 25; Stranger I = 182.85 ± 8.97<br>*D4 25*; Stranger II = 224.90 ± 18.74		*t* = 1.747	0.1112
					D4 50; Stranger I = 194.75 ± 9.39<br>*D4 50*; Stranger II = 230.04 ± 23.08		*t* = 1.350	0.1936
Open field	5 min	Time in center (sec)	VE = 13<br>D4 10 = 15<br>D4 25 = 13<br>D4 50 = 10		*VE* = 37.51 ± 2.02<br>*D4 10* = 41.17 ± 1.08<br>*D4 25* = 29.83 ± 1.03<br>*D4 50* = 37.21 ± 1.03	One−way ANOVA with Bonferroni correction test	$F_{3,50}$ = 0.5989	0.6185
		Number of entries into center			*VE* = 11.57 ± 0.75<br>*D4 10* = 14.60 ± 1.08<br>*D4 25* = 13.00 ± 0.60<br>*D4 50* = 12.38 ± 1.03		$F_{3,50}$ = 1.149	0.3379
		Moving distances (cm)			*VE* = 2298.30 ± 68.81<br>*D4 10* = 2689.70 ± 111.37<br>*D4 25* = 2898.12 ± 132.16<br>*D4 50* = 2712.99 ± 137.22		$F_{3,50}$ = 7.702	0.0002
Forced swim	5 min	Immobility time (sec)	VE = 20<br>D4 10 = 15<br>D4 25 = 11<br>D4 50 = 10		*VE* = 218.35 ± 9.76<br>*D4 10* = 229.94 ± 7.40<br>*D4 25* = 267.38 ± 9.63<br>*D4 50* = 252.42 ± 9.63	One−way ANOVA with Bonferroni correction test	$F_{3,52}$ = 9.217	< 0.0001

Marble burying	30 min	Number of marbles buried (n)	VE = 7<br>D4 10 = 8<br>D4 25 = 7<br>D4 50 = 7		*VE* = 5.28 ± 1.02<br>*D4 10* = 9.88 ± 1.18<br>*D4 25* = 10.14 ± 1.52<br>*D4 50* = 11.40 ± 2.20	One–way ANOVA Bonferroni correction test	$F_{3,25}$ = 5.414	0.0061
Spontaneous self-grooming	10 min	Immobility time (sec)	VE = 10<br>D4 10 = 9<br>D4 25 = 9<br>D4 50 = 8		*VE* = 23.71 ± 4.40<br>*D4 10* = 22.30 ± 2.39<br>*D4 25* = 18.42 ± 5.35<br>*D4 50* = 20.92 ± 3.97	One–way ANOVA with Bonferroni correction test	$F_{3,32}$ = 0.4932	0.6904
Nesting	12h	Nesting score	VE = 10<br>D4 10 = 10<br>D4 25 = 10<br>D4 50 = 10		*VE* = 4.89 ± 0.13<br>*D4 10* = 3.75 ± 0.57<br>*D4 25* = 3.50 ± 0.75<br>*D4 50* = 4.00 ± 0.45	One–way ANOVA with Bonferroni correction test	$F_{3,32}$ = 2.506	0.0787

VE: Vehicle; D4 10: D4 10 mg/kg/day; D4 25: D4 25 mg/kg/day; D4 50: D4 50 mg/kg/day.
